# Emotion dysregulation in adults with attention deficit hyperactivity disorder: a meta-analysis

**DOI:** 10.1186/s12888-020-2442-7

**Published:** 2020-03-12

**Authors:** Ashkan Beheshti, Mira-Lynn Chavanon, Hanna Christiansen

**Affiliations:** Department of Psychology, Child and Adolescent Clinical Psychology Group, Marburg University, Gutenbergstr.18, 35037 Marburg, Germany

**Keywords:** ADHD, Emotion dysregulation, adults, Meta-analysis

## Abstract

**Background:**

Emotional symptoms are increasingly considered a core feature of attention deficit/hyperactivity disorder (ADHD). We aimed to quantify the evidence of emotional dysregulation and its respective facets in individuals with adult ADHD compared to healthy controls using meta-analysis.

**Methods:**

Two electronic databases (PubMed, PsycINFO) were reviewed to identify studies. Studies were eligible for inclusion that had reports on any measure of emotion (dys) regulation in adults (> 18 years of age) in clinically diagnosed patients with ADHD as well as healthy control participants. We included a total of 13 studies (*N* = 2535) to assess (1) the standardized mean difference in emotion dysregulation (ED) as a general factor and its specific facets (i.e., emotional lability, negative emotional responses, and emotion recognition) between adults with ADHD and healthy controls; and (2) the association between ADHD symptom severity and ED.

**Results:**

Compared to healthy controls, adults with ADHD revealed significantly higher levels of general ED (Hedges’ g = 1.17, *p* < 0.001; Hedges’ g is the adjusted effect size). With regard to intermediate dimensions of ED, emotional lability exhibited the strongest weighted effect (Hedges’ g = 1.20, CI [0.57, 1.83], *p* < 0.001). Furthermore, symptom severity and general ED correlated significantly (r = 0.54, *p* < 0.001). Regarding intermediate dimensions of ED, negative emotional responses correlated closely with ADHD symptom severity (r = 0.63, *p* < 0.001) and emotional lability (r = 0.52, *p* < 0.001).

**Conclusions:**

Our findings support ED symptoms as a core feature of ADHD’s psychopathology. With respect to dimensions of ED, emotional lability, and negative emotional responses play a more definitive role in the psychopathology of adults with ADHD. Due to insufficient statistical reports in the included studies, we could not perform meta-regressions to control the role of moderator variables.

## Background

Attention Deficit Hyperactivity Disorder (ADHD) is characterized by its core symptoms inattention, impulsivity, and hyperactivity [1]. The past decade of research revealed that ADHD persists into adulthood [2–4]. Apart from the core symptoms, emotion regulation contributes independently to functional impairments in patients with ADHD [5–7]. In this regard, several studies reported that emotion dysregulation (ED) (subsuming symptoms like low frustration tolerance, irritability, ease of negative emotional experience, and emotional lability) is highly frequent in children, adolescents, and adults with ADHD ([8]; meta-analysis by [9] and qualitative reviews by [10, 11]). About 70% of adult patients with ADHD report ED or emotional lability [5, 8, 12]. Furthermore, ED also exists in patients with ADHD not suffering from any other comorbid mental disorder [8]. Those findings reveal ED as a core component of the disorder or at least as a substantial feature in a subgroup of patients with ADHD (e.g., [4, 13]).

Two decades after Wender [14] recognized features of ED as part of the clinical presentation of adult ADHD, DSM-5 refrains from including such symptoms as indicative of the disorder. Instead, the DSM-5 recommends considering ED as an associated feature of ADHD supporting its diagnosis [1]. According to Kring and Sloan [15], such a limitation occurred due to the fact that ED is still a transdiagnostic concept and can be applied to psychopathological aspects of various disorders not limited to ADHD. Although focusing on emotion regulation and dysregulation might provide a) new insights into the underlying pathophysiological mechanisms (e.g., Shushakova, Ohrmann & Pedersen, [16], b) a more accurate differentiation of symptoms and disorders (e.g., oppositional defiant disorder or conduct disorder vs. ADHS), and c) novel treatment approaches [17–19], research on ED still lacks a consensual and refined definition and depiction of ED and related constructs in general (e.g., [20, 21]), and theoretical frameworks and conceptual models of ED in ADHD in particular. Terms like ED, emotional lability, emotional instability (i.e., irregular shifting between emotional states) and emotional impulsivity (i.e., overshooting emotional responses) are often applied interchangeably or rather idiosyncratically (for a review see [17]). This lack of consensus and clarity regarding the construct of emotion regulation and ED makes summarizing and integrating empirical findings in ADHD complicated [22]. To avoid working in “conceptual and definitional chaos” ([23], p. 330), we briefly define emotion regulation, ED, and facets of ED that contribute to functional and psychosocial impairments in patients with ADHD.

Emotion regulation includes all processes that unfold over time and are related to the different emotions people have, the intensity of emotions, and how emotions are experienced and expressed [24]. The major function of ER is to shape emotional states to facilitate adaptive, goal-directed behavior in a certain situation. The most prominent model of emotion regulation is the modal model [24] that proposes five types of emotion-regulation strategies [25]: (1) taking steps to influence which situation one will be exposed to (situation selection); (2) changing relevant aspects of the situation (situation modification); (3) influencing which portions of the situation are perceived and attended to (attentional deployment); (4) altering the way of thinking about it (reappraisal); and (5) directly modifying emotion-related actions (response modulation). In order to apply such emotion regulation strategies, emotions need to be recognized (i.e., perception and awareness of the self and other’s verbal and nonverbal emotions) [19]. Furthermore, with respect to dysfunctional ER Ryckaert et al. [22] consider all processes that are impaired or fail to modify emotions.

Among those studies and reviews reporting on ED in ADHD, there are at present one systematic review [19] and one meta-analysis [9], both focusing on ED in childhood ADHD. The overview by Shaw et al. [19] summarizes the debate of conceptualizing ED with respect to ADHD by considering ED as a core yet distinct feature that correlates with ADHD. The meta-analysis by Graziano and Garcia [9] analyzed features of ED in children with ADHD. Distinguishing the dimensions of ED in children with ADHD, they demonstrated that such patients are more likely to experience intense emotions. The authors reported that this association between emotional reactivity and the ADHD symptom burden becomes stronger with age, a finding consistent with published reports acknowledging that ED’s impairment persists over the life-span [19, 26, 27].

Relying on the ED facets derived by Graziano and Garcia [9] for children and adolescents, the goal of the present study was to conduct a meta-analysis continuing their work for adult patients with ADHD, as this has not been done so far. As previous empirical work on ADHD symptoms suggests there are differences in symptoms and their trajectories from childhood to adulthood [28–30], this might apply to ED and ED facets as well. We therefore first aimed to identify features of ED in adult ADHD with a literature review. Second, a meta-analysis was conducted to examine a) the magnitude of the associations between ADHD status (patient with ADHD vs. healthy control), ED and its facets; b) the magnitude of the associations between ADHD symptom scores, ED and its facets.

## Methods

### Literature search

This study has been recorded in the international prospective register of systematic reviews (Prospero) in April 2017 with the registration number CRD42017059710. A systematic literature search was undertaken using the electronic databases PubMed and PsychINFO. The literature search was consistent with the ‘Preferred Reporting Items for Systematic Reviews and Meta-Analyses’ (PRISMA) statement [31] and was terminated in December 2019. The Boolean expression used for our search is:

[ADHD* OR “attention deficit hyperactivity disorder” OR ADD* OR “attention deficit disorder” OR hyperkinetic*] AND [“emotion dysregulation” OR “emotion regulation” OR “mood regulation” OR “mood dysregulation” OR “affect regulation” OR “affect dysregulation” OR emotion OR labil*] AND [adult*].

### Inclusion and exclusion criteria

Our search in PubMed yielded 1316 and in PsycINFO 714 abstracts. We also checked the reference lists of included studies for other studies eligible for inclusion. After removing duplicates, abstracts of all articles were screened based on pre-defined inclusion criteria independently by the first author. Inclusion criteria were: (i) report of any self- or third-party measure of emotion, affect, or mood (dys) regulation or emotional lability, (ii) inclusion of clinical samples of adults (> 18 years of age) with ADHD characterized by clinical criteria (e.g., DSM, ICD) and diagnostic procedures, (iii) inclusion of non-ADHD healthy controls. Exclusion criteria were: case reports, conference abstracts, reviews, duplicates and non-English studies. We included only randomized case-control studies that were published in peer-reviewed journals at any time from the inception of the databases. We limited our search to published studies to ensure a level of methodological adequacy and rigor among included studies and to avoid the inevitable problems with securing access to a full set of unpublished studies and the bias that would introduce [32].

**Fig. 1:**
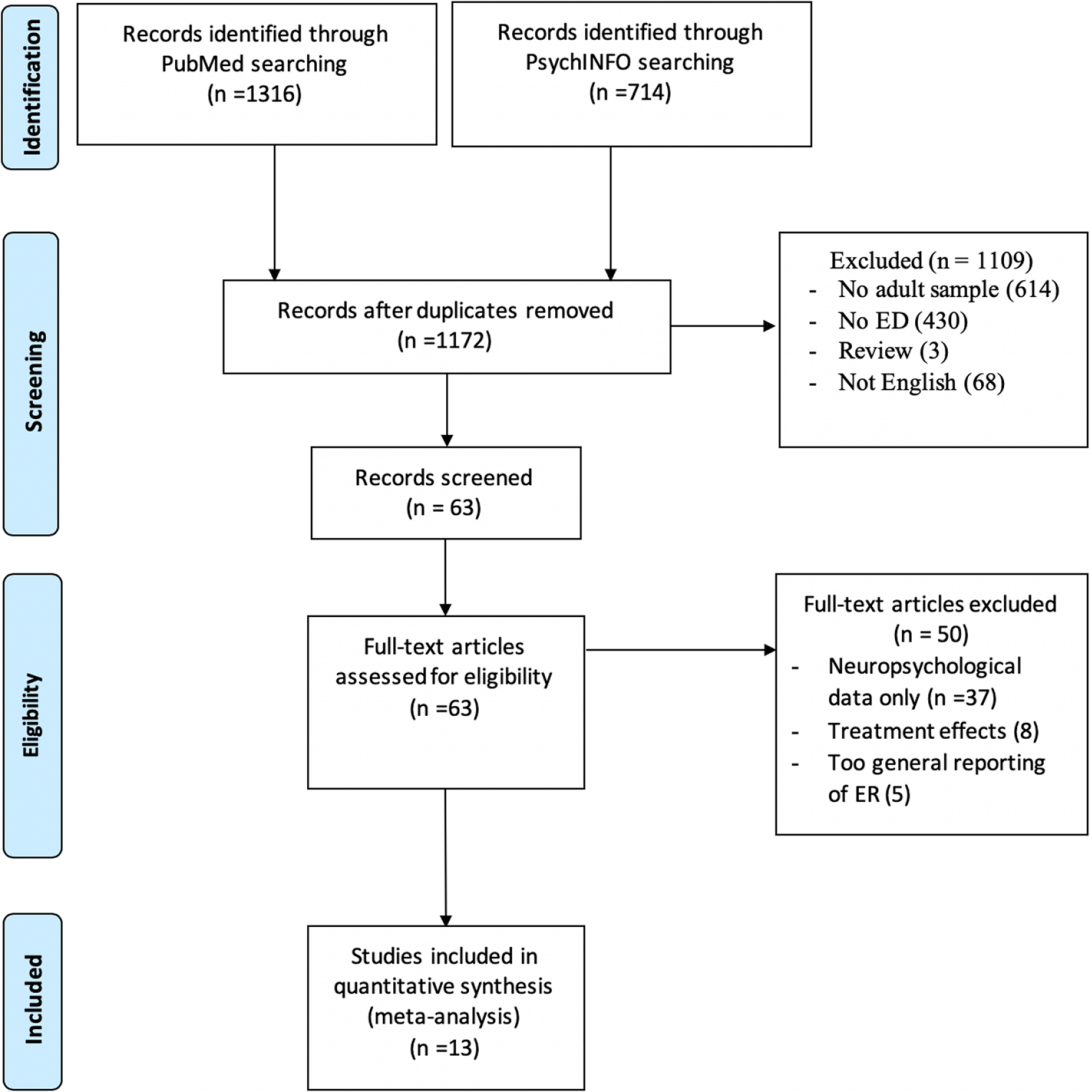
Systematic Search of the Literature: PRISMA Flow-Chart

After scanning a total number of 2030 studies in order to remove the duplicates, 858 studies failed to be included. In the next step, the abstracts of the studies were checked to clarify whether the subject matter is proper to our Boolean expressions. In this step, 1109 studies were excluded since the theme of ADHD in adulthood and measurements of ED were absent. In addition, studies in language other than English as well as reviews were excluded. Next, all titles fulfilling our inclusion criteria (63 studies) were reviewed in full-text. Data were collected and extracted by two independent reviewers that included ADHD status and diagnostic procedures, emotion regulation/dysregulation as defined above, gender composition (male, female), age, comorbidity, and country. When disagreement arose, reviewers consulted with each other until coming to a consensus. Ultimately, 13 studies remained for data extraction (see Fig. 1 for a flow chart of the search).

### Coding and data extraction

In order to code the studies, after the final scanning, three main dimensions of ED were identified based on the narrative synthesis of the literature: Emotion recognition, emotional lability and negative emotional responses. Emotion recognition refers to the perception and awareness of the self and other’s verbal and nonverbal emotions; emotional lability points to an unstable shifting between states of emotions; negative emotional responses refer to irritability and impulsivity of the emotional reactions [19]. For each study and in addition to information on demographics: our statistical results relied on total ED and its extracted facets (emotion recognition, emotional lability or negative emotional responses). Finally, we differentiated the included studies in two parts: the first concerned studies that examined ED between groups with and without ADHD (related to the study’s question of magnitude of the associations between ADHD status, ED and its facets), and the second concerned studies that investigated ED within the groups with ADHD (related to the study’s question of magnitude of the associations between ADHD symptom scores, ED and its facets).

Authors who reported ED but who had not provided enough quantitative data (e.g., only a graphic illustration) were contacted in order to request the necessary information to derive effect size estimates and confidence limits on the selected indices. When only the standard error of the mean (SEM) was reported, the standard deviation (SD) was calculated by multiplying the SEM by the square root of the sample size [33]. When descriptive statistics were reported other than the mean, SD or SEM, data were imputed by established procedures where possible [34].

### Effect estimation and heterogeneity

True effect estimates were computed as adjusted standardized mean differences (Hedges’ g). Meta-analysis was carried out using random-effects models and the results are reported and graphically displayed, as that better conveys data variability [33]. To estimate the average effect size, Hedges’ g criteria were adopted: small = 0.2, medium = 0.5 and large ≥0.8 [35]. Furthermore, as two studies had such small samples (*n* < 20), effect sizes were also calculated with a correction factor converting Cohen’s d to Hedges’ g.

Moreover, to calculate the effect sizes based on correlations, each correlation factor (r) was converted to Fischer’s z. Finally, to report all of a study’s effect sizes in a corresponding unit, Fischer’s z was converted to Hedges’ g.

To examine the consistency of results and estimate to what degree the calculated average effect sizes of a given study are representative, Q and I^2^ statistics were calculated, in which the adopted interpretation amounts are: zero or small heterogeneity for 0 -40%, medium heterogeneity for 40 -70% and high heterogeneity for 70 -90% [36].

To run all the above-mentioned analyses and demonstrate results via forest plots, we carried out initial calculations using Cochrane RevMan 5 and then repeated the calculations using Meta-Mar (1.1.0), a free online meta-analysis service developed by the first author of this study (Beheshti, in preparation).

## Results

### Summary of systematic review

Our systematic literature search revealed thirteen qualifying studies. We used ten of them ([8, 37–47], to run our between-group analysis, as they reported their data for both groups with ADHD and healthy controls. Furthermore, four studies [38, 41, 45, 48] were included to run our within-group analysis, as they only reported data on clinical groups. Moreover, with respect to the identified dimensions of ED, Bodalski, Knouse & Kovalev [47],Cavelti et al. [46], Corbisiero et al. [38], Irastorza [39], Reimherr et al. [48] and Surman et al. [38] reported overall measures of ED. Cavelti et al. [46] and Irastorza & Bellon [49] additionally provided information on the specific facets of negative emotional responses and emotion recognition. The measures used by Bisch et al. [37], Miller et al. [41] and Rapport et al. [50] match the facet of emotion recognition. Mitchell et al. [41], Richard-Lepouriel et al. [45] and Skirrow & Asherson [8] operationalized ED by using scales that assessed the facets of emotional lability and negative emotional responses. Rüfenacht et al., (2019) evaluated all the three dimensions of negative emotional responses, emotion recognition and emotional liability in addition to a total assessment of ED.

Moreover, and with respect to ED measurements, Cavelti et al. [46], Irastorza & Bellon [49] and Mitchell et al. [41] adopted the long version of the Conners’ Adult ADHD Rating Scale self-report that contains a subscale on Impulsivity and Emotional Lability, and used the Emotion Regulation Skills Questionnaire to measure emotion regulation skills. Furthermore, Bodalski, Knouse & Kovalev [47] and Irastorza & Bellon [49] employed the Deficient Emotional Self-Regulation (DESR) scale additionally, which is a section of the self-report Current Behavior Scale developed by Barkley [51] for assessing ED. Reimherr et al. [48] and Corbisiero et al. [38] assessed ED via the Affect Lability, Temper and Emotional Overreactivity subscales of the Wender-Reimherr Adult Attention Deficit Disorder Rating Scale. Miller et al. [40] and Rapport et al. [50] administered the Diagnostic Assessment of Nonverbal Accuracy (DANVA [52];) as an assessment to identify facial emotional expression. In addition, Rapport et al. [50] and Richard-Lepouriel et al. [45] administered The Affect Intensity Measure (AIM; [53] to examine experienced aspects of emotion. Richard-Lepouriel et al. [45] and Skirrow & Asherson [8] also employed the Self-rated Affective Lability Scale (ALS [54]) to measure emotional lability and negative emotional responses. Bisch et al. [37] employed the Self-Report Emotional Intelligence Test (SREIT, [55]) to measure the ability to recognize, manage, and engage in one’s own and others’ emotions. Surman et al. [44] used the Deficient Emotional Self-Regulation scale (DESR [51];) to measure ED (see Table 1 for details). Rüfenacht et al. [43] administered The Emotion Reactivity Scale (ERS, [56]) which consists of three subscale of emotion sensitivity, intensity and persistence in order to evaluate ED.

**Table 1:** Summary of studies and calculated effect sizes

Study	Age(M ± SD)	sample size	Measurements of ED	ED dimensions	design	Effect size
[37]	28.52 ± 8.53	54	The Self-report of Emotional Intelligence Test	ER	Between	0.70
[47]	30.47 ± 9.20	159	DERS	Total ED	Between	0.92
[46]	33.39 ± 9.4	135	Impulsivity/Emotional Lability scale from the Conners’ CAARS	Total ED, ER, NE	Between	1.67
[38]	32.27 ± 10.98	514	Emotional Dysregulation Derived from the Wender-Reimherr Adult Attention Deficit Disorder Rating Scale	Total ED	Between Within	2.04 1.38
[49]	36.29 ± 10.71	105	Impulsivity/Emotional Lability scale from the Conners’ CAARS, DERS	Total ED, ER, NE	Between	0.85
[40]	33.82 ± 9.90	51	Diagnostic Analysis of Nonverbal Accuracy	ER	Between	0.30
[41]	23.58 ± 5.31	41	Impulsivity/Emotional Lability scale from the Conners’ CAARS	EL, NE	Between Within	0.95 2.10
[50]	34.85 ± 11.20	56	Affect Intensity Measure, Diagnostic Analysis of Nonverbal Accuracy	ER	Between	0.25
[48]	41.20 ± 11.20	536	Emotional Dysregulation Derived from the Wender-Reimherr Adult Attention Deficit Disorder Rating Scale	Total ED	Within	1.09
[45]	38.14 ± 11.43	198	Affective Lability Scale, Affect Intensity Measure	EL, NE	Between Within	0.98 1.31
[43]	35.49 ± 12.86	366	ERS	Total ED, EL, NE, ER	Between	0.59
[8]	28.76 ± 9.98	88	The Affective Lability Scale-Short Form	EL, NE	Between	1.87
[44]	28.42 ± 8.78	232	self-report Current Behavior Scale developed by R. Barkley	Total ED	Between	2.71

*ED* Emotion Dysregulation, *ER* Emotion Recognition, *EL* Emotional Lability, *NE* Negative Emotions. Between: comparison between ADHD and control group, within: association of ADHD symptoms with emotion dysregulation within the ADHD group

Regarding the studies’ results, Bodalski, Knouse & Kovalev [47], Cavelti et al. [46], Corbisiero et al. [38], Irastorza [39], Reimherr et al. [48], Rüfenacht et al. [43] and Surman et al. [44] reported a significant difference between ED scores (regardless of its specific dimensions) of the groups with ADHD and healthy controls (*p* < 0.01, *p* < 0.01, *p* < 0.001, *p* < 0.01, *p* < 0.01, *p* < 0.001 and *p* < 0.01, respectively). Cavelti et al. [46], Irastorza & Bellon [49] and Rüfenacht et al., [43] also reported a strong association between negative emotional responses and emotion recognition in their ADHD group (*p* < 0.01, for all of them). Moreover, the studies by Bisch et al. [37], Miller et al. [40], Rapport et al. [50] and Rüfenacht et al. [43] demonstrated a distinct difference between groups regarding the facet of emotion recognition (*p* < 0.01 for all of them). In addition, emotional lability and negative emotional responses were significantly associated in patients with ADHD in investigations by Mitchell et al. [41], Richard-Lepouriel et al. [45], Rüfenacht et al. [43] and Skirrow & Asherson [8] (*p* < 0.01, for all of them).

Furthermore, the study by Corbisiero et al. [38] was the only one we included that investigated comorbidity as a moderating variable. In this context, they observed a significant difference between ADHD + ED with comorbidity and ADHD + ED with no comorbidity (*p* < 0.01). Also, the study by Cavelti et al. [45] was the only one in which ED was not only investigated in differences between patients with ADHD and healthy controls, but also it compared ED in ADHD with ED in another mental disorder: they found that patients with ADHD and borderline personality disorder exhibit significantly higher levels of emotional lability than a healthy group (*p* < 0.001). However, the difference in emotional lability was not significant between patients with ADHD and borderline personality disorders (*p* = 0.81). Table 1 provides an overview of the studies included in this meta-analysis.

### Summary of the meta-analysis

#### ED differences between patients with ADHD and control groups

**Fig. 2:**
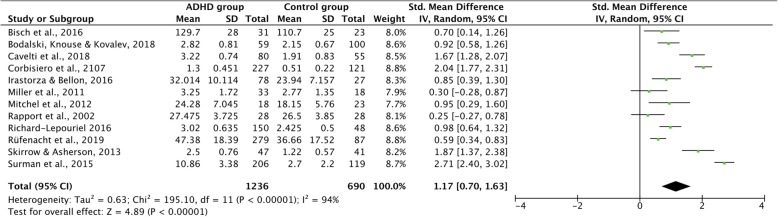
Meta-Analysis Forrest Plot (random-model analysis) comparing ED in ADHD and healthy controls

In answering our study’s first question, namely whether groups with and without ADHD differ in emotion regulation, we noted a large average effect size of *g =* 1.17 (95% CI [0.70, 1.64], *p* < 0.001) for general emotion dysregulation according to the random effects model (for details see Fig. 2 and Table 2). In addition, with respect to specific dimensions, medium to large effect sizes were revealed for emotional lability (*g =* 1.20 (95% CI [0.57, 1.83], *p* < 0.001), negative emotional responses (*g =* 1.12 (95% CI [0.57, 1.68], *p* < 0.001), and emotion recognition (*g =* 0.63 (95% CI [0.40, 0.85], *p* < 0.001). However, results of an analysis of the variance ANOVA showed that the difference between those specific dimensions was not significant (F = 1.33, ns).

**Table 2:** Effect Sizes for differences in ED dimensions between adults with and without ADHD

	ED	ER	EL	NE
Hedges’g	1.17	0.63	1.20	1.12
95% CI	[0.70,1.64]	[0.40, 0.85]	[0.57, 1.83]	[0.57, 1.68]
Hedges’g Criteria	Large	Moderate	Large	Large
I^2^	94%	40%	90%	91%
Criteria of I^2^	High	Moderate	High	High
Number of studies	12	6	4	6
Number of participants	1926	767	695	933

*ED* Emotion Dysregulation, *ER* Emotion Recognition, *EL* Emotional Lability, *NE* Negative Emotions. I^2^: Heterogeneity of the study

#### Association of ED with severity of ADHD symptoms

**Fig. 3:**
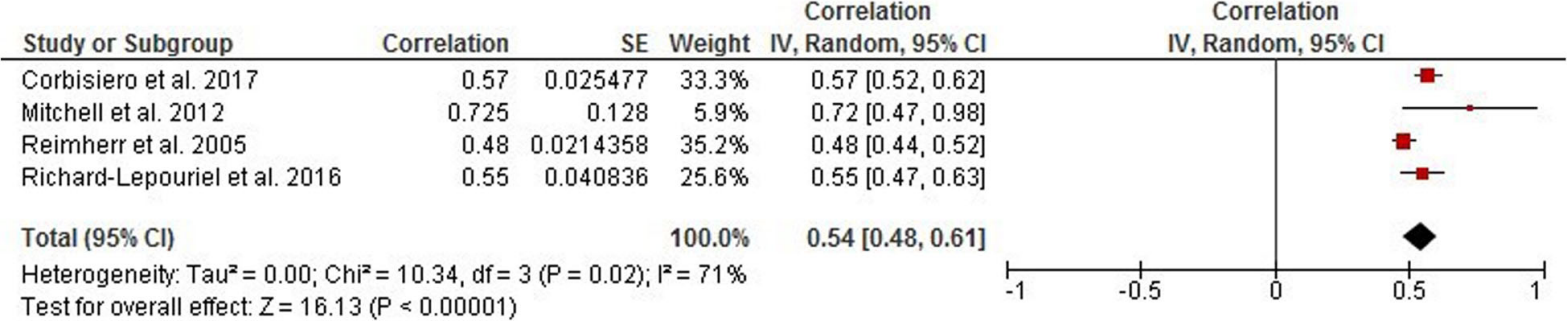
Meta-Analysis Forrest Plot on Correlation Coefficients between measures of ADHD symptom severity and ED

Answering our study’s second question regarding a correlation between ADHD symptoms in adults and emotion dysregulation dimensions, we found a strong correlation between the severity of ADHD symptoms and ED in general with an average effect size of *r = 0.54* (95% CI [0.48, 0.61], *p* < 0.001; for details see Fig. 3 and Table 3). However, our data on the correlation between the severity of ADHD symptoms and specific ED dimensions revealed that negative emotional responses contribute more with a weighted effect of *r = 0.63* (95% CI [0.30, 0.99], *p* < 0.001) whereas emotional lability revealed a slightly smaller weighted effect of *r = 0.52* (95% CI [0.31, 0.73], *p* < 0.001). However, results of an analysis of the variance (ANOVA) showed that the difference between those specific dimensions was not significant (F = 0.27, ns).

**Table 3:** Effect sizes for differences in ED dimensions in adults with ADHD

	ED	EL	NE
Correlation Coefficient (r)	0.54	0.52	0.63
95% CI (random-model)	[0.48, 0.61]	[0.31, 0.73]	[0.30, 0.99]
r criteria	Large	Large	Large
I^2^	71%	54%	68%
Criteria of I^2^	High	Medium	Medium
Number of studies	4	2	2
Number of samples	1097	168	168

*ED* Emotion Dysregulation, *ER* Emotion Recognition, *EL* Emotional Lability, *NE* Negative Emotions. I^2^: Heterogeneity of the study

#### Heterogeneity of analysis

I^2^ values are presented in Tables 2 and 3. The total heterogeneity of ED’s average effect size in between-group studies was 94% and for emotion recognition, emotional lability, and negative emotional responses 40, 90 and 91%, respectively. In the within-group studies, the total heterogeneity of the average effect size was 71%, and for emotional lability and negative emotional responses 54 and 68%, respectively.

**Fig. 4:**
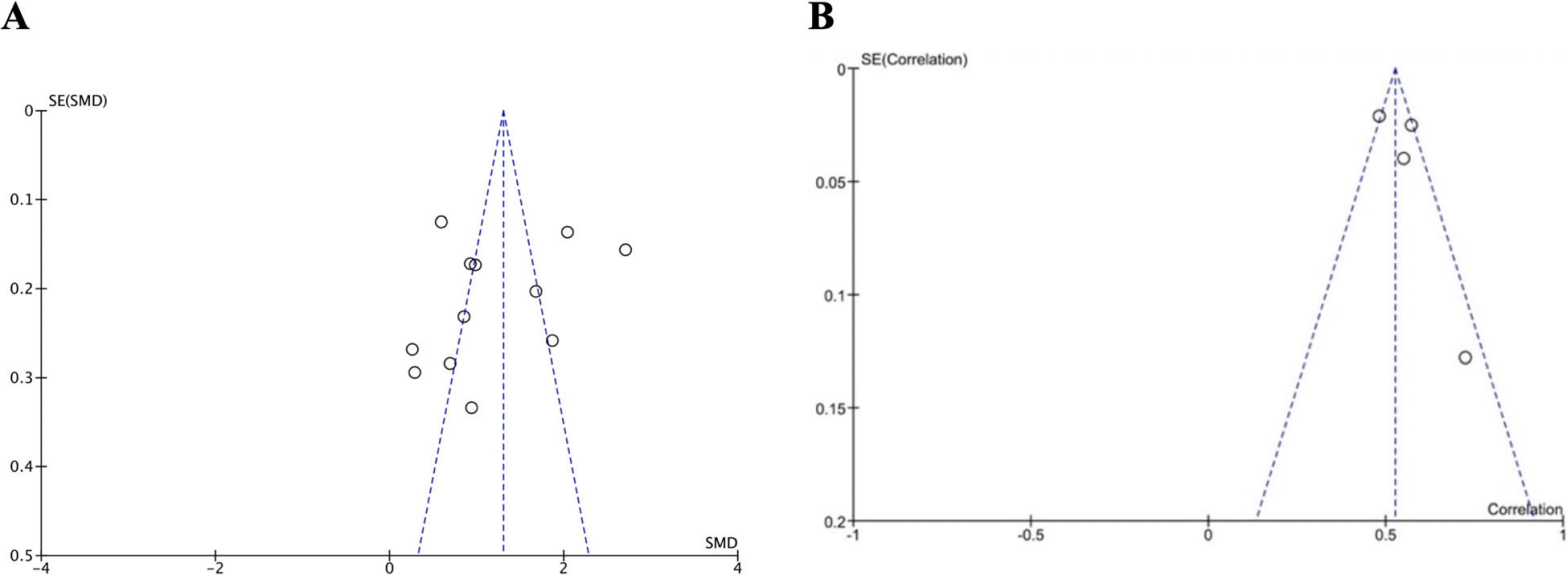
Funnel Plot **a**) refers to the meta-analysis comparing ED in ADHD and healthy controls **b**) refers to the meta-analysis of the correlation coefficients between measures of ADHD symptom severity and ED

To control for any analysis bias, we used funnel plots and Fail-safe N tests. Our between-group analysis results showed that the funnel plot is asymmetric, with the smaller studies tending toward the left of the average effect size. This may indicate that there are studies missing from the right side. Consequently, were there no such probable bias, the average effect size could be larger than the aforementioned amount. In addition, our Fail-safe N test results showed that 1481 studies need to be added to our analysis to reduce the effect size to statistical non-significance (for details see Fig. 4a).

Concerning our within-group analysis, we noted an asymmetric funnel plot with the smaller studies leaning to the right of the average effect size. Four studies were included at this level with sample sizes of 539, 396, 150 and 18 ([38, 41, 45, 48]), respectively. This may indicate studies missing from the left side. Therefore, the average effect size might be smaller than the current estimate. In addition, our Fail-safe N test results showed that 732 studies need to be added to our analysis to reduce the effect size to statistical non-significance (for details see Fig. 4b).

## Discussion

The present meta-analysis was conducted to establish aspects of ED in adulthood ADHD and to differentiate such aspects between ADHD and healthy control groups. Another goal of our study was to assess any association between ED’s features and ADHD symptoms. In line with these research objectives, we identified dimensions of ED based on our adopted conceptual models of ED (i.e., emotion regulation model by Gross [57], and regarding features of ED in adulthood ADHD that focused on the studies we selected for this study. Three dimensions of emotion recognition (ER), emotional lability (EL), and negative emotional responses (NE) were distinguished.

We then categorized the 13 studies selected (*N* = 2535) by two labels of between-group studies (10), in which data was reported on both groups with ADHD and health controls, and within-group studies (4), in which only data on patients with ADHD was available. At the between-group analysis level, we found that compared to a control group, emotion dysregulation is significantly more pronounced in adults with ADHD with a large effect size (Hedges’ g = 1.17). Furthermore, regarding ED’s intermediate dimensions, emotional lability revealed the largest effect size (Hedges’ g = 1.20). Previous studies demonstrated the relevance of ED for mental and somatic health in general (e.g., [58, 59]) and for ADHS in particular [19]. Barkley & Fischer [60] demonstrated that adult patients with persisting ADHD reported worse ED than healthy control participants. In another example, Corbisiero et al. [7] differentiated affective lability from reactivity and temper as core features of ED; they reported higher rates of ED in adults with ADHD. Compatible with these findings, our results support the significant difference between the rates of ED in adulthood ADHD and control groups. Skirrow & Asherson [8] also reported that emotional lability contributed independently to impairing the daily life of adults with ADHD.

Finally, we observed a strong correlation between the severity of ADHD symptoms and ED (r = 0.54). In terms of ED, dimensions, negative emotional responses exhibited the strongest correlation with the core ADHD symptoms (r = 0.63) - findings that concur with the literature [16, 26, 61], and that are also compatible with the study by Graziano & Garcia [9] that reported a stronger correlation between emotional responses and ADHD symptoms in older adolescents.

Our results demonstrate that emotional lability plays both a significant role in differentiating clinical groups with ADHD from healthy controls and a strong correlation between negative emotional responses and ADHD symptom severity. Referring to adopted conceptual models, these findings might be explained by considering the following: First, the literature suggests that concerning ADHD’s epidemiology in adults, the evidence that hyperactive-impulsive symptoms seem to remit in older age groups may be attributable to adaptive strategies patients develop over the life-span, while inattention symptoms seem to persist [62–64];). In this regard, these symptoms might be correlated better with impaired situation identification that requires attention processes, as well as a lack of strategies for monitoring emotion regulation processes – which in turn would trigger higher rates of emotional lability in adults with ADHD. Second, the severity of ADHD symptoms in adults correlated significantly with negative externalizing behaviors such as aggression and irritation, as articulated by Posner et al. [65] in their *dyscontrol hypothesis* and *affectivity hypothesis*. Based on dyscontrol hypothesis, impairments in the capacity of inhibiting the emotional responses occurs significantly more in hyperactive subgroups of ADHD. In this regard, functional neuroimaging shows anomalies within frontolimbic circuits. According to the affectivity hypothesis, negative-emotionally-responsive behavior in ADHD patients emerges through the route of dysfunctional emotional processing associated with the amygdala and medial prefrontal cortex [65]. Emotion recognition seems to be a more serious problem in young people with ADHD. As the Graziano & Garcia [9] analysis implied, emotion recognition skills are weaker in younger children, and as cognitive abilities develop and adapt, emotion recognition improves. Therefore, although emotion recognition remains a feature of ED in adulthood ADHD, emotional lability and negative emotional responses play a more pronounced role in the ED-associated psychopathology of adults with ADHD.

### Practical implications of the results

Most likely, ED in adults with ADHD is a problem persisting from childhood that either was addressed with no specific intervention (as juvenile ADHD treatment is predominantly pharmacological [66, 67];)), or that was therapy-resistant and continues to be a obvious feature over the course of the disorder [68, 69]. In this regard, our study findings support the consideration of therapeutic approaches entailing ED improvement strategies and reinforcing emotion regulation skills, in addition to standard interventions for the disorder [4, 70]. Furthermore, as our results demonstrate, such therapeutic strategies and interventions would be advisable to focus on a general ED impairment in adults with ADHD while considering emotional lability and negative emotional responses as aspects of ED that need to be targeted in adults with ADHD. Moreover, there is strong evidence of the effectiveness of pharmacological [27, 71, 72];) and psychotherapeutic interventions in alleviating emotion dysregulation and the disorder’s core symptoms [4, 6, 11, 73, 74]. In light of the problem of diagnosing ADHD in adulthood and the lack of specific criteria for adults, our meta-analysis findings suggest that adopting an approach that addresses aspects of ED in the diagnosis and treatment of adults with ADHD would yield a valuable supplementary benefit.

### Study limitations

We assumed that different measures of ED are contingent, though that might not always be the case. As the adopted conceptual models of ED converged in the studies included in our meta-analysis, we did not analyze them accordingly.

Moreover, reviews have shown that moderators such as gender and cognitive functions ([75–77]; as well as the presence of comorbidity [3] or medication [6] play a significant role in ADHD. Therefore, if emotion dysregulation is assumed to be a main feature of ADHD in adults, controlling for such moderators should be part of a meta-analysis. However, as the studies included contained a paucity of such statistical data, we could not perform meta-regressions that might have shed light on such moderators.

Finally, our funnel plots and Fail-safe N test results imply that (probably) missing studies and t hus omitted from our meta-analysis contributed to asymmetrically distributed effect sizes. In between-group analysis, missing studies would strengthen, and in within-group analysis weaken effects. In addition, the lesser degree of heterogeneity (71%) in our within-group analysis compared to the between-group analysis might be a sample-size problem.

## Conclusions

In conclusion, our results from the present meta-analysis focusing on the role of emotion dysregulation in adulthood ADHD imply that compared to a control group, ED is a distinct feature of adult ADHD. Furthermore, the severity of ADHD symptoms significantly correlates with dimensions of ED such as emotional lability, emotion recognition, and emotional responses, replicating other studies in the field (e. g., [78]). In addition, classic domains of inattention, hyperactivity, and impulsivity do not sufficiently explain the entire symptom spectrum. In this respect, assessing and targeting emotion regulation in clinical practice might prove to be a valuable strategy for diagnosing and treating adult ADHD. Moreover, future research should clarify how ED interacts with adult ADHD symptoms, comorbid conditions, and other moderators such as demographics.

## Data Availability

The datasets used and analyzed during the present study are available from the corresponding author on reasonable request.

## Abbreviations

ADHD: Attention deficit/hyperactivity disorder
AIM: Affect Intensity Measure
ALS: Affective Lability Scale
DANVA: Diagnostic Assessment of Nonverbal Accuracy
DESR: Deficient Emotional Self-Regulation
ED: Emotion dysregulation
EL: Emotional lability
ER: Emotion recognition
ERS: Emotion Reactivity Scale
NE: Negative emotional responses
SD: Standard deviation
SEM: Standard error of the mean
SREIT: Self-Report Emotional Intelligence Test
